# Sulfotransferase 1A1 (*SULT1A1*) gene expression is regulated by members of the NFI transcription factors in human breast cancer cells

**DOI:** 10.1186/1472-6890-14-1

**Published:** 2014-01-06

**Authors:** Aiwei Yao-Borengasser, Lora J Rogers, Vineetha K Edavana, Rosalind B Penney, Xinfeng Yu, Ishwori B Dhakal, Suzanne Williams, Susan A Kadlubar

**Affiliations:** 1 Division of Medical Genetics, College of Medicine, University of Arkansas for Medical Sciences, 4301 West Markham St., Little Rock, AR, 72205, USA

**Keywords:** SULT1A1, NFI, Human breast cancer cell lines, Gene expression regulation, siRNA transfection, Gene copy number

## Abstract

**Background:**

Sulfotransferase 1A1 (*SULT1A1*) gene expression is tissue specific, with little to no expression in normal breast epithelia. Expression in breast tumors has been documented, but the transcriptional regulation of *SULT1A1* in human breast tissue is poorly understood. We identified Nuclear Factor I (NFI) as a transcription factor family involved in the regulation of SULT1A1 expression.

**Methods:**

Transcription Factor Activation Profiling Plate Array assay was used to identify the possible transcription factors that regulate the gene expression of *SULT1A1*in normal breast MCF-10A cells and breast cancer ZR-75-1 cells. Expression levels of NFI-C and SULT1A1 were determined by real-time RT-PCR using total RNA isolated from 84 human liver samples. Expression levels of SULT1A1, NFI-A, NFI-B, NFI-C, and NFI-X were also determined in different human breast cancer cell lines (MCF-7, T-47D, ZR-75-1, and MDA-MB-231), in the transformed human epithelial cell line MCF-10A, and in ZR-75-1 cells that were transfected with siRNAs directed against NFI-A, NFI-B, NFI-C, or NFI-X for 48 h. The copy numbers of *SULT1A1* in cell lines ZR-75-1, MCF-7, T-47D, MDA-MB-231, and MCF-10A were determined using a pre-designed Custom Plus TaqMan^®^ Copy Number kit from Life Technologies.

**Results:**

In normal human liver samples, SULT1A1 mRNA level was positively associated with NFI-C. In different human breast cancer and normal epithelial cell lines, SULT1A1 expression was positively correlated with NFI-B and NFI-C. SULT1A1 expression was decreased 41% and 61% in ZR-75-1 cells treated with siRNAs against NFI-A and NFI-C respectively. SULT1A1 gene expression was higher in cells containing more than one *SULT1A1* copy numbers.

**Conclusions:**

Our data suggests that SULT1A1 expression is regulated by NFI, as well as *SULT1A1* copy number variation in human breast cancer cell lines. These data provide a mechanistic basis for the differential expression of SULT1A1 in different tissues and different physiological states of disease.

## Background

Treatment of estrogen receptor- (ER-) positive breast cancer with tamoxifen (TAM) has been the gold standard for the past 30 years [1]. TAM is also the only FDA-approved breast cancer chemoprevention agent, as its use is associated with decreased occurrence of contralateral breast cancer in patients treated with adjuvant TAM [2]. Both the therapeutic efficacy and adverse effects of TAM vary considerably among individuals [3]. In terms of therapeutic efficacy, 4-hydroxy-TAM and endoxifen are the major active metabolites of TAM. The detoxification of 4-hydroxy-TAM is carried out by phase II enzymes such as sulfotransferases, including SULT1A1 and uridine diphosphate glucuronosyltransferase (UGTs) [4].

Sulfotransferases catalyze the transfer of a sulfonate group from 3′-phosphoadenosine 5′ -phosphosulfate (PAPS) to a range of xenobiotic and endogenous compounds such as drugs, carcinogens, and steroid hormones [5]. The human SULT1A subfamily consists of SULT1A1, SULT1A2, SULT1A3, and SULT1A4. SULT1A1 protein is found in many different human tissues, with the highest abundance found in the liver [6-11]. SULT1A1 activity varies several-fold among individuals [12-14]. It has been shown that gene expression/protein activity levels of SULT1A1 affect the efficacy of TAM treatment [15-18]. SULT1A1 can also contribute to increased cancer risk (as reviewed in [19]), including breast cancer risk [20-22]. Furthermore, SULT1A1 expression is related to disease state, with little to no expression in normal breast epithelia but plentiful protein expression in most breast tumors [23,24]. Given the role that SULT1A1 plays in drug efficacy and in individual susceptibility to disease, it is important to elucidate the factors regulating the differential expression of SULT1A1.

Some studies have shown that single nucleotide polymorphisms (SNPs) in the human *SULT1A1* promoter, 3′-untranslated region (UTR), and coding regions contribute to SULT1A1 availability and activity [13,25-27]. However, SNPs account for only a small percentage of the variation of SULT1A1 activity. Some studies demonstrated that *SULT1A1* gene copy number variants (CNV) exist in human populations, and that CNV is associated with SULT1A1 activity [13,28,29]. Nevertheless, doubling *SULT1A1* copy number does not appear to double its activity, indicating that factors other than copy number are determinants of SULT1A1 activity. Thus, other factors must play a role in regulating the expression of SULT1A1. Traditionally, SULT1A1 has been considered a non-inducible enzyme. Since its expression is variable across tissues, it could be postulated that transcription factor (TF) binding (and the tissue-specific availability of TFs) may affect gene expression in SULT1A1. There have been reports of TF regulation of SULT1A1. There is one report that demonstrated that *SULT1A1* promoter activity is dependent on the presence of ubiquitous Ets family transcription factors [30]. While these TFs appear to be responsible for basal, constitutive expression, TFs that affect differential SULT1A1 gene expression have not been examined. In this study, TFs differentially expressed between low SULT1A1-expressing transformed epithelial mammary cells and high SULT1A1-expressing breast cancer cells have been identified for the first time using a TF Activation Profiling Plate Array assay.

## Methods

### Cell culture

The human breast cancer cell lines ZR-75-1, MCF-7, T-47D, MDA-MB-231, and the human transformed mammary epithelial cell line MCF-10A were purchased from American Type Culture Collection (Rockville, MD) and were sustained in a 37°C incubator containing 5% CO_2_. ZR-75-1 cells were cultured in RPMI 1640 medium with 10% fetal bovine serum (FBS). MCF-7 cells were cultured in Improved MEM medium with 10% FBS and 0.01 mg/ml insulin (Life Technologies, Carlsbad, CA). MDA-MB-231 cells were cultured in DMEM medium with 10% FBS. MCF-10A cells were cultured in DMEM/F12 medium supplemented with 5% chelex-treated horse serum (Life Technologies), 20 mg/ml of epidermal growth factor (Life Technologies), 10 μg/ml insulin (Sigma, St. Louis, MO), 0.5 g/ml hydrocortisone (Sigma), and 0.1 μg/ml cholera toxin (Sigma).

### Human subjects

Normal human liver samples (n = 84) were obtained from the U.S. Cooperative Human Tissue Network under a University of Arkansas for Medical Sciences Institutional Research Committee-approved protocol. All specimens were snap-frozen upon collection. Normal tissue status was confirmed with histology by the Cooperative Human Tissue Network. Samples included 33 women and 51 men, all of whom were Caucasian. The average age of the study population was 59, ranging from 26 to 102.

### Differential transcription factor activation assay

The differential TF activation profile in ZR-75-1 and MCF-10A cells was determined using a TF Activation Profiling Plate Array assay following the manufacturer’s instructions (Signosis, Sunnyvale, CA). Briefly, 5 μg of nuclear extract from ZR-75-1 or MCF-10A cells was hybridized with 48 different biotin-labeled probes that contain consensus sequences of TF DNA-binding sites. The TF-bound probes were captured by complementary sequences of the probes pre-coated on a 96-well plate. The captured probes were detected with streptavidin-HRP. The levels of luminescence of each corresponding well were quantitatively analyzed with Spectramax M5 (Sunnyvale, CA) and compared between MCF-10A cells and ZR-75-1 cells.

### Nuclear factor I (NF-I) siRNA treatment of ZR-75-1 cells

All siRNAs and transfection reagents were purchased from Life Technologies. ZR-75-1 cells that were at the exponential growth stage were transfected with 50 pmol of pre-designed siRNA against 4 NFI family members (NFI-A, NFI-B, NFI-C, NFI-X) or 100pmol siGAPDH as a negative control using Lipofectamine™ 2000 transfection reagent following the manufacturer’s instructions. After 48 h transfection, cells were collected for total RNA isolation. The percentage of knockdown of target gene expression was determined using quantitative RT-PCR (qRT-PCR). The sequences of siRNAs are listed in Table 1.

**Table 1:** Primer sequences

**Gene**	**Sequences (forward)**	**Sequences (reverse)**
18S	5′ TTCGAACGTCTGCCCTATCAA 3′	5′ ATGGTAGGCACGGCGACTA 3′
GAPDH	5′ ACAGTCAGCCGCATCTTCTT 3′	5′ ACGACCAAATCCGTTGACTC 3′
NFI-A	5′ CCAGCGCCCGGCAGTTATGT 3′	5′ ATTCATCCTGGGTGAGACAGAGCGG 3′
NFI-B	5′ AACCAGCCAGCCTAACGGCA 3′	5′ TCGCACTGCACTGGGATGGG 3′
NFI-C	5′ GACATGGAAGGAGGCATCTC 3′	5′ GGGCTGTTGAATGGTGACTT 3′
NFI-X	5′ CCACTGCCCAACGGGCACTTA 3′	5′ CCGTCACATTCCAGACCCCGGA 3′
SULT1A1	5′ AGGAGTTCATGGACCACAGC3′	5′ TGAAGGTGGTCTTCCAGTCC3′
siNFI-A	5′ GGUAUUCCGCUGGAAAGUAtt 3′	
siNFI-B	5′ AGUGUCAUCUCAACUCGAAtt 3′	
siNFI-C	5′ GGACAGGGCGUCUUCCUAAtt 3′	
siNFI-X	5′ GAAUCCGGACAAUCAGAUAtt 3′	

### DNA and RNA isolation and quantitative real-time PCR (qRT-PCR)

Total RNA and total DNA from cultured cells and from snap-frozen human liver tissues were isolated with an AllPrep^®^ DNA/RNA/protein kit (QIAGEN, Valencia, CA) according to the manufacturer’s instructions. The quantity and quality of the isolated RNA and DNA were determined by an Agilent 2100 Bioanalyzer (Agilent Technology, Palo Alto, CA). qRT-PCR using SYBR-green reagent was performed and analyzed as described previously[31]. qRT-PCR using TaqMan^®^ reagents (Life Technologies) was carried out as detailed previously [13]. The primer sequences used with SYBR-green reagent are listed in Table 1.

### Determination of *SULT1A1* gene copy number

*SULT1A1* gene was amplified by relative quantitative PCR using a pre-designed Custom Plus TaqMan^®^ Copy Number kit following the company’s instructions (Life Technologies). *SULT1A1* copy number variations were calculated using the Applied Biosystems CopyCaller™ software (Life Technologies).

### Statistical analysis

Paired *t* tests were used to compare baseline and treatment measurements within a group. Pearson’s correlation coefficients were used to describe the linear association between variables. All data from samples were expressed as mean ± sem.

## Results

### SULT1A1 Expression levels in different breast cancer cell lines

**Figure 1:**
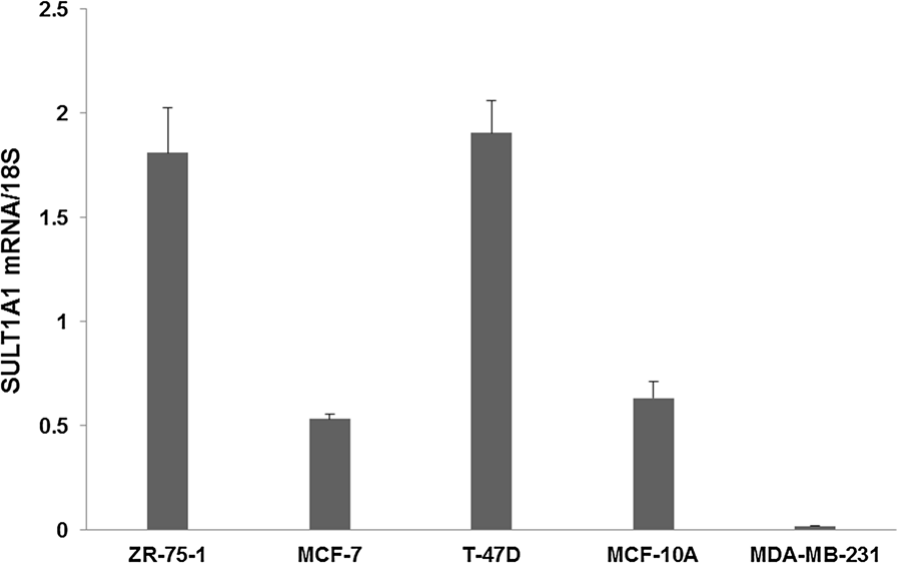
**SULT1A1 mRNA levels in different cell lines.** Total RNA was isolated from cells that were cultured at exponential growth stage. The mRNA levels of SULT1A1 in ZR-75-1, MCF-7, T-47D, MCF-10A, and MDA-MB-231 were determined by real-time RT-PCR and were normalized with 18S.

Other laboratories have reported that expression of SULT1A1 is very low in normal breast tissue as compared to tumor tissue [23,24]. To test if this is also true in cultured cells, the mRNA levels of SULT1A1 were determined in the breast cancer cell lines ZR-75-1, MCF-7, T-47D, MDA-MB-231 and the human transformed breast epithelial cell line MCF-10A with relative qRT-PCR. Data were analyzed as absolute quantification and expressed in relation to 18S RNA. Standard curves were generated using pooled cDNA from the samples assayed. Figure 1 shows that ZR-75-1 cells and T-47D cells had higher SULT1A1 mRNA levels compared to the other cell lines. Compared to the phenotypically normal epithelial MCF-10A cells, SULT1A1 expression was more than 3 times higher in ZR-75-1 and T-47D cells. MCF-7 cells showed similar expression levels of SULT1A1 as MCF-10A cells. SULT1A1 was not expressed in MDA-MB-231 cells.

### Correlation of gene expressions between NFI family genes and SULT1A1

**Figure 2:**
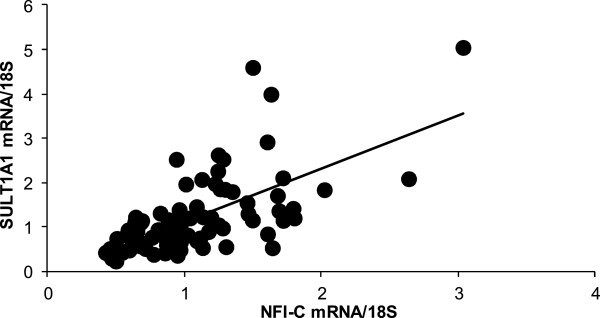
**Correlation between SULT1A1 mRNA and NFI-C mRNA in Human liver.** SULT1A1 mRNA level was highly correlated with NFI-C mRNA level (r = 0.64, p < 0.0001). Real-time RT-PCR was performed in liver samples taken from 84 healthy subjects as described in Methods. Data was normalized with 18S.

One possible mechanism of differential SULT1A1 expression in different cell lines could be the availability of TFs. To test this hypothesis, the TF Activation Profiling Plate Array assay was performed in normal breast MCF-10A cells and breast cancer ZR-75-1 cells. TF activation profile differences in MCF-10A cells and ZR-75-1 cells were quantitatively analyzed and compared as described in Methods. Out of 48 TFs, 13 had at least 1.5 fold more DNA-binding activity in ZR-75-1 cells compared to MCF-10A cells (data not shown, see Additional file 1). After matching the 13 TFs to the TF binding consensus sequences in SULT1A1 using Transcriptional Regulatory Element Database software (Cold Spring Harbor, NY), NFI and GATA transcription factor 1 (GATA-1) were chosen as the transcription factors most likely to regulate SULT1A1 expression. SULT1A1 promoter contains a GATA-1 binding site (CCTGCCTATC) at position -367 bp and a NFI binding site (TGTTGGCTGC) at position -298 bp. There are four genes encoding NFI in humans, namely *NFI-A, NFI-B, NFI-C,* and *NFI-X*. To determine the relationship between NFI or GATA-1 and SULT1A1, mRNA levels of each were measured using total RNA isolated from 84 normal human liver samples. As seen in Figure 2, TaqMan^®^ qRT-PCR results demonstrate that NFI-C (chosen as a representative form of NFI) mRNA levels were significantly correlated with SULT1A1 mRNA levels (r = 0.64, p < 0.0001). GATA-1 mRNA level, on the other hand, showed no association with SULT1A1 expression level (r = 0.17, p = 0.11, see Additional file 2).

Next, gene expression correlations between SULT1A1 and all four individual NFI family genes in different cell lines were explored. mRNA levels of NFI-A, NFI-B, NFI-C, NFI-X, and SULT1A1 in MCF-10A, ZR-75-1, T-47D, MCF-7, and MDA-MB-231 were determined by qRT-PCR and normalized with 18S mRNA. SULT1A1 gene expression was highest in ZR-75-1 and T-47D cells, as were NFI-B and NFI-C gene expression (Table 2). Pearson’s correlation coefficients were used to describe the linear association between SULT1A1 and NFI family gene expressions. SULT1A1 mRNA levels were highly correlated across cell lines with NFI-B (r = 0.95, p < 0.01) and NFI-C (r = 0.97, p < 0.01). SULT1A1 mRNA levels showed a positive correlation trend with NFI-A (r = 0.54) and NFI-X (r = 0.65) even though the correlation coefficients did not reach statistical significance (Table 2).

**Table 2:** Correlation of SULT1A1 mRNA level with NFI-A, NFI-B, NF-C, and NFI-X, mRNA levels in different cell lines

	**NFI-A/18S**	**NFI-B/18S**	**NFI-C/18S**	**NFI-X/18S**	**SULT1A1/18S**
ZR-75-1	0.81	2.17	1.44	1.65	2.11
T-47D	4.08	2.55	1.43	0.93	1.74
MCF-7	0.04	0.21	0.70	0.36	0.54
MCF-10A	1.09	0.32	0.63	1.12	0.57
MDA-MB-231	0.47	0.08	0.57	0.83	0.00
	**Correlation coefficients of SULT1A1 mRNA with NFI family gene expressions (n = 5)**				
	**NFI-A**	**NFI-B**	**NFI-C**	**NFI-X**	**SULT1A1**
Correlation coefficients	0.53	0.95	0.97	0.65	1.00
p value	>0.1	<0.01	<0.01	>0.05	

### Regulation of SULT1A1 gene expression by NFI family genes

**Figure 3:**
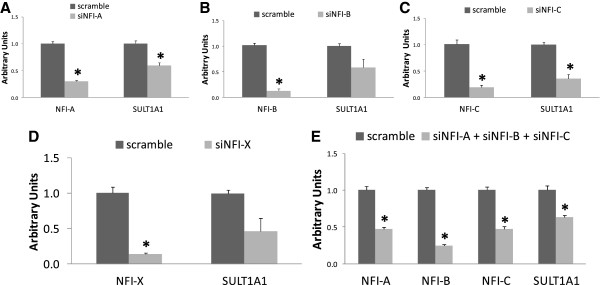
**Effects of NFI siRNAs on SULT1A1 mRNA gene expression.** ZR-75-1 cells were treated with siRNA against NFI mRNAs as described in Methods. Total RNA was extracted for gene expression analysis 48 h later (n = 3 for each mRNA expression level). Data was normalized with 18S. *p < 0.05. **A:** SULT1A1 expression in siNFI-A treated cells. **B:** SULT1A1 expression in siNFI-B treated cells. **C:** SULT1A1 expression in siNFI-C treated cells. **D:** SULT1A1 expression in siNFI-X treated cells. **E:** SULT1A1 expression in co-transfected siNFI-A, siNFI-B, and siNFI-C cells.

To determine if any of the four NFI genes has an effect on SULT1A1 gene expression, ZR-75-1 cells, which have the highest SULT1A1 gene expression in this study, were transfected with siRNAs that were directed against NFI-A, NFI-B, NIF-C, or NFI-X for 48 h as described in Methods. The mRNA levels were determined by relative RT-PCR, and the 2^-ΔΔCT^ method was used [31]. As shown in Figure 3, mRNA levels of NFI-A (A), NFI-B (B), NFI-C (C), and NFI-X (D) were reduced by 70% ± 0.02, 87% ± 0.08, 81% ± 0.08, and 86% ± 0.12, respectively. SULT1A1 gene expression was significantly decreased by 41% ± 0.05 (p = 0.05) in siNFI-A (A) transfected cells and 64% ± 0.08 (p = 0.05) in siNFI-C (C) transfected cells. Although not statistically significant, SULT1A1 mRNA levels were also decreased in siNFI-B (B) (41% ± 0.16, p = 0.07) and siNFI-X (D) transfected cells (53% ± 0.18, p = 0.15). Further examination revealed that SULT1A1 gene expression was decreased more than four-fold in siNFI-B transfected MCF-7 cells (data not shown, see Additional file 3).

To test whether different NFI TFs had an additive effect on regulating SULT1A1 expression, ZR-75-1 cells were also co-transfected with siNFI-A, siNFI-B, and siNFI-C for 48 h. As shown in Figure 3E, SULT1A1 mRNA level was decreased about 40%, which is similar to that of each individual NFI mRNA knockdown. Hence, the results indicated that there was no additive transcriptional regulatory effect on SULT1A1 expression by those three NFI TFs.

### Relationship of SULT1A1 gene expression with its gene copy number

Previous reports demonstrate that individual DNA contains one to five copies of the SULT1A1 gene [28]. To determine if CNV is also evident in different cell lines, the CNV of *SULT1A1* was determined in ZR-75-1, MCF-7, T-47D, MDA-MB-231, and MCF-10A cell lines. *SULT1A1* copy numbers were calculated and normalized to an endogenous reference gene RNase P, known to be present in two copies in a diploid genome. Table 3 details the results of the calculated *SULT1A1* copy numbers in the cell lines tested. Genomic DNA of MCF-7, MDA-MB-231, and MCF-10A contains only one copy of *SULT1A1*, while ZR-75-1 DNA has 3 copies and T-47D DNA has 5 copies of *SULT1A1*.

**Table 3:** Gene copy numbers and mRNA Levels

**Cell line**	**MCF-7**	**MDA-MB-231**	**MCF-10A**	**ZR-75-1**	**T-47D**
**Copy number**	**1**	**1**	**1**	**3**	**5**
SULT1A1 mRNA/18S	0.58 ± 0.03	0.00	0.75 ± 0.08	2.24 ± 0.21	2.17 ± 0.26
NFI-A mRNA/18S	0.06 ± 0.08	0.75 ± 0.00	0.93 ± 0.00	0.49 ± 0.00	4.48 ± 0.10
NFI-B mRNA/18S	0.22 ± 0.01	0.16 ± 0.01	0.19 ± 0.01	1.95 ± 0.17	3.51 ± 0.29
NFI-C mRNA/18S	0.62 ± 0.02	1.15 ± 0.05	0.80 ± 0.03	0.90 ± 0.02	1.64 ± 0.02
NFI-X mRNA/18S	0.49 ± 0.03	1.32 ± 0.7	1.14 ± 0.11	1.35 ± 0.16	1.08 ± 0.09

*SULT1A1* copy numbers and gene expression levels of SULT1A1, NFI-A, NFI-B, NFI-C, and NFI-X were determined in different cell lines.

To test if copy numbers correlate with SULT1A1 gene expression level, the amount of mRNA of SULT1A1 and corresponding *SULT1A1* copy numbers were compared in each cell line. The levels of mRNA were determined by RT-PCR using a standard curve generated from pooled cDNA samples from different cell lines. The data was normalized with 18S and expressed as a ratio. As shown in Table 3, cell lines with one copy of *SULT1A1* (MCF-7 and MCF-10A) had lower gene expression, while cell lines with more than one copy number (ZR-75-1 and T-47D) had higher SULT1A1 gene expression. ZR-75-1 cells expressed similar amount of SULT1A1 mRNA as T-47D, even though ZR-75-1 DNA has two fewer copies of SULT1A1 than T-47D cells. Although MDA-MB-231 DNA contains one copy of SULT1A1, there was no SULT1A1 gene expression detected.

## Discussion

SULT1A1 is expressed in different tissues, in different physiological states of the same tissue, and in different individuals. The factors that regulate SULT1A1 gene expression are poorly understood. Human studies show that SULT1A1 can be regulated by alternative promoter usage [32], SNPs in the coding or promoter region [26,27], CNVs [28], or variants in the 3′UTR of the gene [13]. However, these genetic variants account for only a portion of the variation of SULT1A1 activity. Hempel *et al*. [30] identified Ets synergized with Sp1 as one of the regulators of SULT1A1 expression. It is postulated that ubiquitous Ets is utilized to ensure constant expression of SULT1A1 in tissues such as liver, skin, and gut, since SULT1A1 is important in xenobiotic metabolism. This current study elucidated NFI gene family as an important TF in regulating SULT1A1 expression in different cell types.

The data presented here suggest that NFI may play a major role in regulating SULT1A1 expression in different physiological and disease states. The NFI family proteins are associated with changes in different cell growth states and with oncogenic processes and disease states as reviewed in [33-35]. TF profiling array data demonstrated that NFI in high-SULT1A1-expressing ZR-75-1 breast cells was expressed more than in low-SULT1A1-expressing non-cancer MCF-10A cells. This positive association was also observed in human liver samples and in different human cell lines. Further siRNA transfection assays suggested that SULT1A1 expression is controlled, at least partially, by NFI in breast cancer cells. While the siRNA data demonstrate NFI-B has the least effect on SULT1A1 expression, there is still an effect and the p-value approached significance at p = 0.07, while the NFI isoforms with significant effects were at p = 0.05. This effect may not be a direct one, and NFI-B may not be the only factor which has an effect on SULT1A1 expression. Rather it may be part of another mechanism that has a compound effect on SULT1A1 expression. This could explain the lower significance of NFI-B. There was no additive SULT1A1 expression inhibition observed when ZR-75-1 cells were co-transfected with siNFI-A, siNFI-B, and siNFI-C, suggesting that each gene has its own role in regulating SULT1A1 expression.

NFI siRNA knockdown experiments inhibited about 40% of SULT1A1 expression in ZR-75-1 cells, suggesting other regulatory mechanism(s) exist. Previous studies have shown that the human population possesses one to five copies of *SULT1A1*, and that its enzyme activity is correlated with CNV [13,14,28]. The data presented here demonstrate that there were *SULT1A1* copy number differences in human cell lines also, and that cells with more copies had higher SULT1A1 expression than cells with only one copy. However, higher copy number does not always correlate with the degree of expression. T-47D cells, which contained five copies, had similar SULT1A1 expression to that of ZR-75-1 cells, which contained three copies. MCF-7 and MDA-MB-231cells each contained one copy, yet MDA-MB-231 cells showed no SULT1A1 expression, while MCF-7 cells showed a modest amount of SULT1A1 expression. The data suggests that SULT1A1 gene expression could also be regulated by mechanisms other than NFI and copy numbers. *SULT1A1* CNV in different cell lines reflect the range of *SULT1A1* CNV reported in human populations [13,14,28]. Thus, cell lines could be used as a model for human *SULT1A1* CNV-related studies.

## Conclusions

In summary, our data demonstrate that the NFI family of transcription factors significantly contributes to the regulation of SULT1A1 expression in human breast cancer cell lines. Along with these TFs, *SULT1A1* CNV determines gene expression. Understanding the regulation of SULT1A1 expression will facilitate the prediction of drug response in relation to breast cancer prevention and therapy.

## Consent

Written informed consent was obtained from the patient for the publication of this report and any accompanying images.

## Abbreviations

SULT1A1: Sulfotransferase 1A; NFI: Nuclear factor I; ESR1: Estrogen receptor α; UTR: Untranslated region; CNV: Copy number variant; UGT: Uridine diphosphate glucuronosyltransferase; PAPS: 3′-phosphoadenosine 5′ –phosphosulfate.

## Competing interests

The authors declare that they have no competing interests.

## Authors’ contributions

SAK and AY-B participated in the study design. AY-B, LJR, RBP, XY, VKE, and SW carried out data collection. AY-B, RJP, VKE, IBD, SAK contributed in data analysis and interpretation. SAK, AY-B, RBP, and VKE were involved in manuscript preparation. All authors have read and approved the final manuscript.

## Pre-publication history

The pre-publication history for this paper can be accessed here:

http://www.biomedcentral.com/1472-6890/14/1/prepub

## Supplementary Material

Additional file 1 **Differential transcription factor activities levels between MCF-10A and ZR-75-1 cells.** Transcription factor activation profile was determined using a TF Activation Profiling Plate Array as described in Methods. Data chosen from results showing that transcription factor activation level in ZR-75-1 cells (black bar) was at least 1.5 fold higher than that in MCF-10A cells (gray bar). Click here for file

Additional file 2 **Correlation between GATA-1 mRNA and NFI-C mRNA in Human liver.** Real-time RT-PCR was performed in liver samples taken from healthy subjects as described in Methods. Data was normalized with 18S. GATA-1 mRNA level was not correlated with SULT1A1 mRNA level (r = 0.17, p = 0.11). Click here for file

Additional file 3 **Screening of SULT1A1 gene expression activators from MCF-7 cells treated with siRNAs.** SULT1A1 mRNA levels were determined with The Expressed Transcription Factor Knockdown Transcriptome PCR Array (Qiagen, Valencia, CA) following the manufacturer’s instructions. Briefly, real-time PCR was performed with cDNAs derived from MCF-7 cells treated with siRNAs targeting 270 transcription factors. SULT1A1 gene expression levels were expressed as Log2 fold changes based on Ct calculation using 18S as house-keeping gene and non-target siRNA treated sample well (VTC) as negative control. The arrow indicates SULT1A1 gene expression was decreased more than four-fold in siNFI-B transfected MCF-7 cells. Click here for file
