# Supplementary material for: Sulfotransferase 1A1 (SULT1A1) gene expression is regulated by members of the NFI transcription factors in human breast cancer cells
Source: BMC Clin Pathol. 2014 Jan 6;14:1. doi: 10.1186/1472-6890-14-1 (PMC3913331; doi:10.1186/1472-6890-14-1)
Supplement: Additional file 1 — Differential transcription factor activities levels between MCF-10A and ZR-75-1 cells. Transcription factor activation profile was determined using a TF Activation Profiling Plate Array as described in Methods. Data chosen from results showing that transcription factor activation level in ZR-75-1 cells (black bar) was at least 1.5 fold higher than that in MCF-10A cells (gray bar). [file 1472-6890-14-1-S1.pdf]

SUPPLEMENTARY FIGURE 1

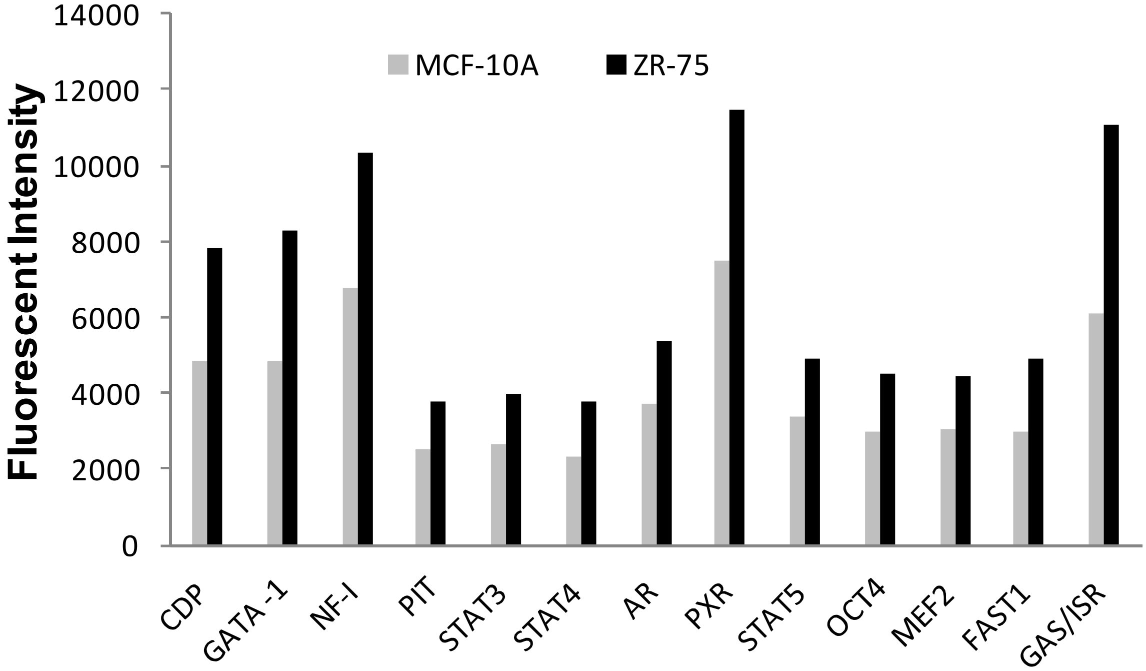

Supplementary Figure 1. Differential transcription factor activities levels between MCF-10A and ZR-75 cells. Transcription factor activation profile was determined using a TF Activation Profiling Plate Array as described in Methods. Data chosen from results showing that transcription factor activation level in ZR-75 cells (black bar) was at least 1.5 fold higher than that in MCF-10A cells (gray bar).
