# Supplementary figures and images for: Sulfotransferase 1A1 (SULT1A1) gene expression is regulated by members of the NFI transcription factors in human breast cancer cells
Source: BMC Clin Pathol. 2014 Jan 6;14:1. doi: 10.1186/1472-6890-14-1 (PMC3913331; doi:10.1186/1472-6890-14-1)

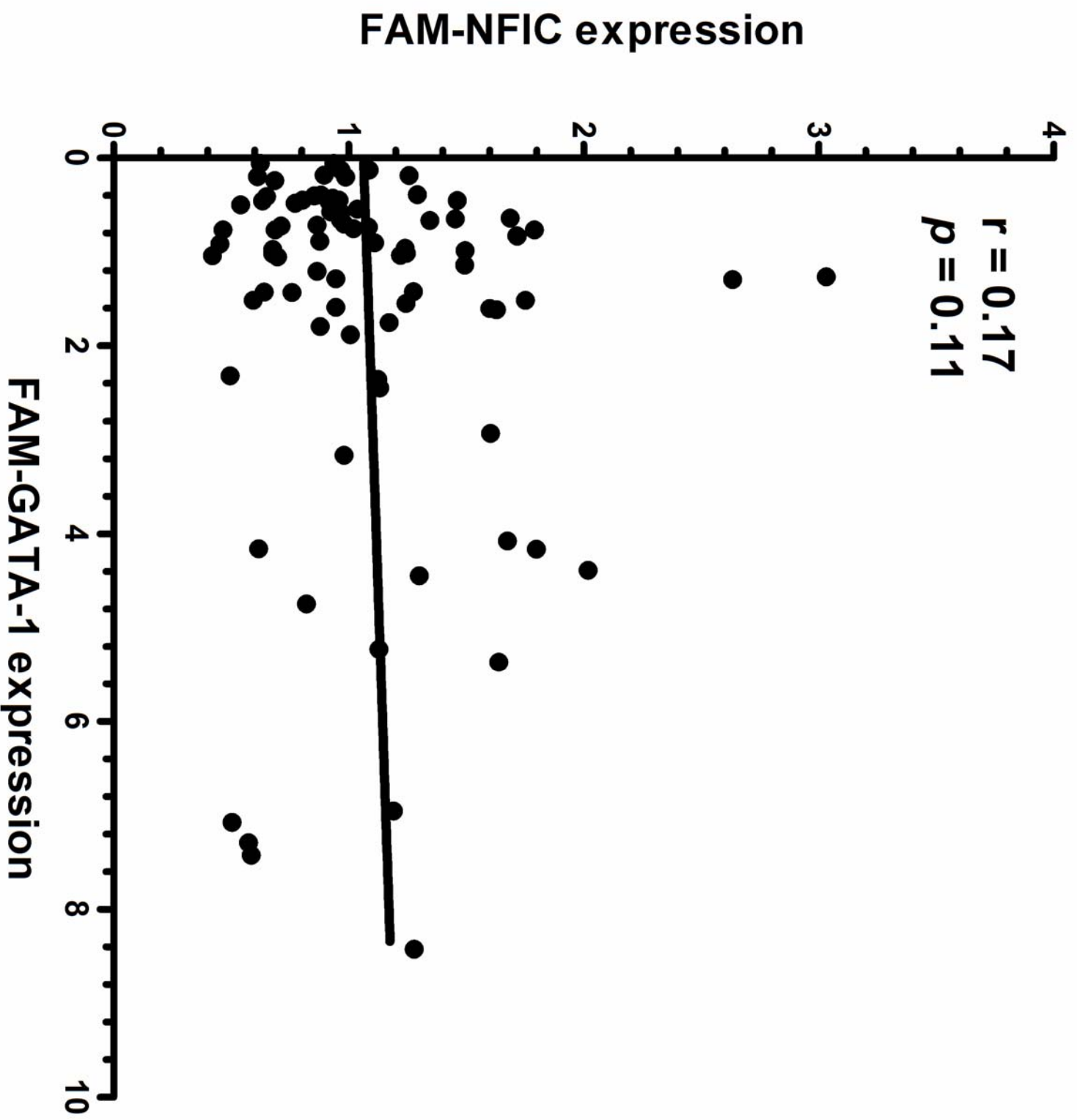

Supplement: Additional file 2 — Correlation between GATA-1 mRNA and NFI-C mRNA in Human liver. Real-time RT-PCR was performed in liver samples taken from healthy subjects as described in Methods. Data was normalized with 18S. GATA-1 mRNA level was not correlated with SULT1A1 mRNA level (r = 0.17, p = 0.11). [file 1472-6890-14-1-S2.pdf]
